# Risk factors of left atrial appendage thrombus in patients with non-valvular atrial fibrillation

**DOI:** 10.1515/med-2021-0009

**Published:** 2021-03-03

**Authors:** Yaowu Liu, Didi Zhu, Yunyun Xiao, Yeqian Zhu, Qianxing Zhou, Liqun Ren, Long Chen

**Affiliations:** Department of Cardiology, Zhongda Hospital of Southeast University, Nanjing, 210009, China; Department of Geriatrics, Affiliated Drum Tower Hospital, Nanjing University Medical School, Nanjing, 210008, China; Department of Cardiology, The First Affiliated Hospital of Nanjing Medical University, Nanjing, 210029, China

**Keywords:** atrial fibrillation, left atrial appendage thrombus, risk factors

## Abstract

**Objective:**

To investigate the risk factors of left atrial appendage thrombus (LAAT) in patients with non-valvular atrial fibrillation (AF).

**Methods:**

We collected the clinical data of patients with non-valvular AF who underwent transesophageal echocardiography (TEE) at the Zhongda Hospital of Southeast University between January 2016 and June 2019. The patients were divided into two groups, LAAT and non-LAAT. We performed comparative analysis, receiver operating characteristic (ROC) curve analysis and logistic regression analysis to estimate the risk factors of LAAT.

**Results:**

A total of 442 patients with non-valvular AF were enrolled in the study. LAAT was detected by TEE in 20 cases (4.7%). Compared with patients without LAAT, patients with LAAT had higher CHA_2_DS_2_-VASc scores (3 vs 2, *p* = 0.001), higher values of D-dimer (180.0 vs 90.0 μg/L, *p* = 0.003), larger LA anteroposterior diameters (50.5 vs 41.0 mm, *p* < 0.001) and higher ratios of non-paroxysmal AF (85.0% vs 23.6%, *p* < 0.001). ROC curve analysis revealed that the cutoff value of LA anteroposterior diameter was 49.5 mm. After adjusting for other confounders, logistic regression analysis showed that enlarged LA (anteroposterior diameter ≥49.5 mm) and non-paroxysmal AF were independently associated with higher risks of LAAT (OR = 7.28, 95% CI: 2.36–22.47; OR = 8.89, 95% CI: 2.33–33.99, respectively). The proportions of LAAT in patients with larger LA (anteroposterior diameter ≥49.5 mm), non-paroxysmal AF and both larger LA and non-paroxysmal AF were 30% (12/40), 15.2% (17/112) and 39.1% (9/23), respectively.

**Conclusion:**

Enlarged LA (anteroposterior diameter ≥49.5 mm) and non-paroxysmal AF were independent risk factors of LAAT in non-valvular AF patients.

## 1 Introduction

Atrial fibrillation (AF) is the most common sustained arrhythmia with a prevalence of approximately 3% in adults [1,2], accounting for one-third of hospitalizations for cardiac rhythm disorders [3]. AF is independently associated with increased risks of mortality and morbidity, partly due to increased risk of stroke caused by the arrhythmia [4,5]. AF-related ischemic stroke is the result of detachment of left atrial thrombus. Studies revealed that 90% of left atrial thrombi originate from left atrial appendage (LAA) [6], and thrombus of LAA was associated with its structure and morphology [7]. In spite of the anatomical factors of LAA, other risk factors associated with left atrial appendage thrombus (LAAT) were rarely reported. Early detection and intervention of LAAT-related risk factors are important for reducing the incidence of thromboembolism in AF patients. Herein, we collected and analyzed the clinical data of patients with non-valvular AF who underwent transesophageal echocardiography (TEE) to explore the potential risk factors of LAAT.

## 2 Methods

### 2.1 Study population

This retrospective cohort study included consecutive patients with non-valvular AF who underwent TEE at the Zhongda Hospital of Southeast University between January 2016 and June 2019. Evaluation of AF was based on diagnostic criteria of the latest guidelines for the management of AF [8]. According to clinical characteristics, AF can be classified into paroxysmal, persistent, or long-standing persistent AF. Paroxysmal AF is defined as AF that terminates spontaneously or with intervention within 7 days. Persistent AF is defined as continuous AF that is sustained beyond 7 days. Long-standing persistent AF is defined as continuous AF of >12 months duration. Since the exactly continuous time of some patients with persistent AF was difficult to ensure, we were unable to distinguish persistent AF from long-standing persistent AF for these cases. In this study, we classified AF into two subtypes, namely, paroxysmal AF (episodes that last for ≤7 days) and non-paroxysmal AF (episodes that sustain for >7 days). We excluded patients with valvular AF (AF patients with moderate to severe mitral stenosis or mechanical heart valves), with congenital heart disease, and who received anticoagulant therapy >3 weeks before TEE tests. This study was approved by the ethics committee review board of Zhongda Hospital of Southeast University, China.

### 2.2 Data collection

We collected general and clinical information, including age, gender, history of smoking, drinking and chronic diseases, CHA_2_DS_2_-VASc score, AF types, results of blood tests, measured parameters of transthoracic echocardiography (TE) and TEE, from medical records in the hospital system. LAAT was defined as abnormal lumpy echogenic masses of LAA seen in ≥2 sections with clear boundaries using TEE [9].

### 2.3 Statistical analysis

Included patients were divided into two groups (LAAT and non-LAAT). Continuous variables were presented as median and quartiles, and non-parametric test was used for inter-group comparisons. Categorical variables were presented as percentages, and *χ* ^2^ test was used for comparisons. The receiver operating characteristic (ROC) curve was used to analyze and determine the appropriate cutoff point of risk factors to predict LAAT. Logistic regression analyses were performed to find independent risk factors of LAAT, and the results were expressed as odds ratios (ORs) with 95% confidence intervals (CIs). All statistical analyses were performed using STATA 12.0 software (Stata Corporation, College Station, TX, USA). All tests were two-sided and *p* < 0.05 was considered significant.

## 3 Results

### 3.1 Clinical characteristics of study population

A total of 422 patients [234 males and 188 females; median age, 65 years (range, 33–86 years)] with non-valvular AF who underwent TEE, were enrolled in the study. TEE revealed LAAT in 20 out of 422 cases (4.7%). Compared with the non-LAAT group, patients in the LAAT group had higher score of CHA_2_DS_2_-VASc (3 vs 2, *p* = 0.001), higher value of D-dimer (180.0 vs 90.0 μg/L, *p* = 0.003), greater anteroposterior diameter of left atrium (LA) (50.5 vs 41.0 mm, *p* < 0.001), and higher proportion of non-paroxysmal AF (85.0% vs 23.6%, *p* < 0.001). However, there were no significant differences in gender, age, smoking, drinking, history of diseases (hypertension, diabetes, coronary heart disease), values of platelets, prothrombin time (PT), activated partial thromboplastin time (APTT), serum creatinine, left ventricular end diastolic dimension (LVEDD) and left ventricular ejection fraction (LVEF) between the two groups. The baseline characteristics of patients are presented in Table 1.

**Table 1:** Comparisons of baseline characteristics between LAAT and non-LAAT groups

Clinical characteristics	LAAT (*n* = 20)	Non-LAAT (*n* = 402)	*P* value
Male, *n* (%)	8 (40)	226 (56.2)	0.154
Age, median (IQR) – years	63 (53–73)	65 (58–72)	0.088
Smoking, *n* (%)	4 (20)	85 (21.1)	0.903
Drinking, *n* (%)	3 (15)	32 (8)	0.265
Hypertension, *n* (%)	16 (80)	246 (61.2)	0.093
Diabetes, *n* (%)	5 (25)	54 (13.4)	0.147
Coronary artery disease, *n* (%)	10 (50)	142 (35.3)	0.185
CHA_2_DS_2_-VASc score, median (IQR)	3 (2–4)	2 (1–3)	0.001
Platelet count, median (IQR), 10^9^/L	174 (152–217)	184 (148–220)	0.641
PT, median (IQR), s	12.2 (11.4–15.5)	12.0 (11.3–13.6)	0.360
APTT, median (IQR), s	32.9 (30.3–41.85)	32.4 (29.6–37.1)	0.324
D-dimer, median (IQR), μg/L	180.0 (69.5–330.1)	90.0 (49.0–142.8)	0.003
SCr, median (IQR), μmol/L	75.0 (61.3–92.5)	77.0 (65.0–88.0)	0.703
LA anteroposterior diameter, median (IQR), mm	50.5 (44.0–54.4)	41.0 (37.4–44.7)	<0.001
LVEDD, median (IQR), mm	47.5 (45.3–50.0)	46.3 (43.3–50.0)	0.250
LVEF, median (IQR)	0.65 (0.58–0.70)	0.67 (0.60–0.71)	0.226
Non-paroxysmal AF, *n* (%)	17 (85.0)	95 (23.6)	<0.001

LAAT, left atrial appendage thrombus; IQR, inter-quartile range; PT, prothrombin time; APTT, activated partial thromboplastin time; SCr, serum creatinine; LA, left atrium; LVEDD, left ventricular end diastolic dimension; LVEF, left ventricular ejection fraction.

### 3.2 Results of ROC curve analysis

**Figure 1:**
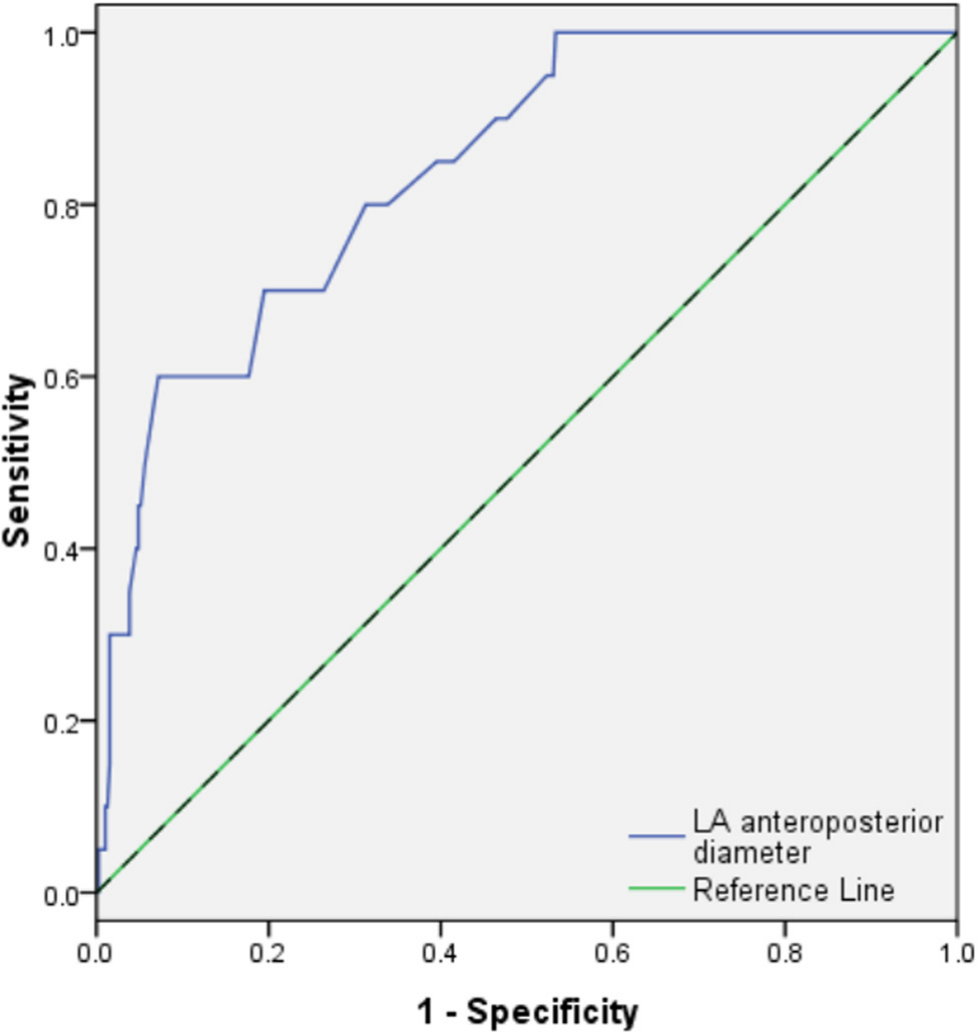
ROC analysis of LA anteroposterior diameter in predicting LAAT. LA = left atrium, LAAT = left atrial appendage thrombus.

**Figure 2:**
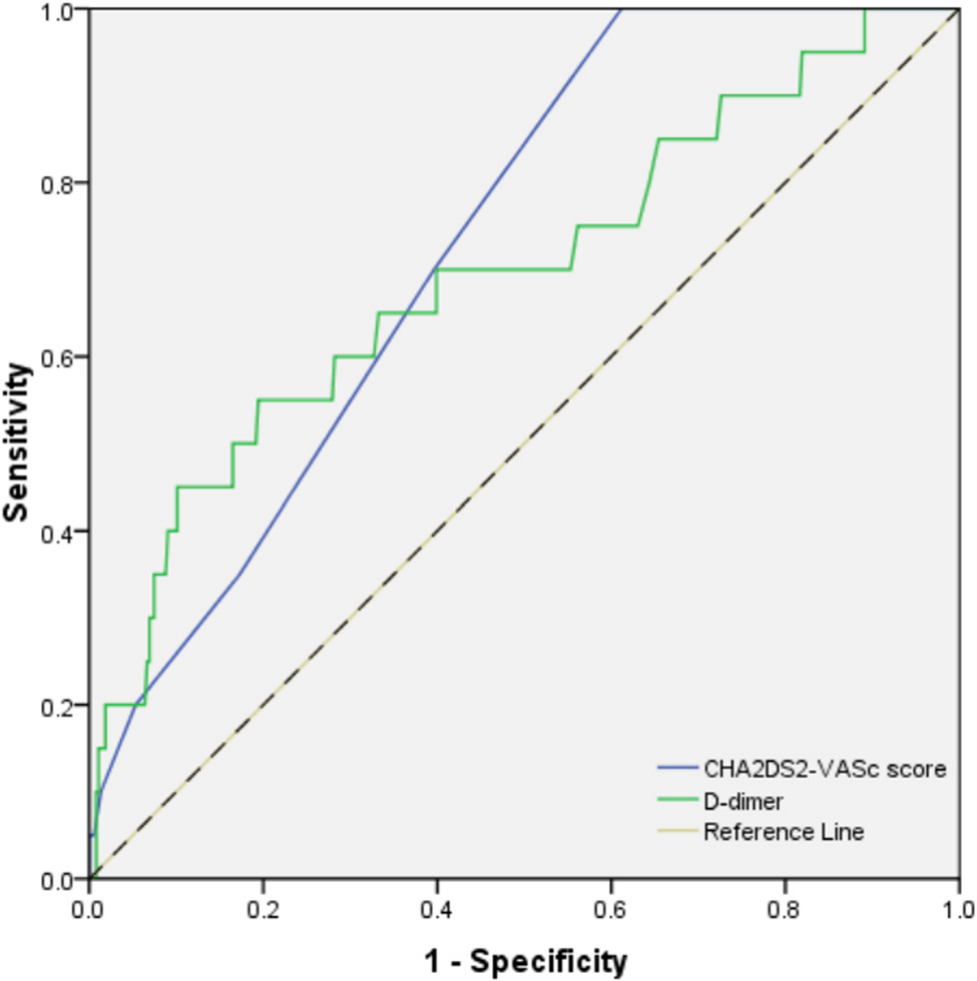
ROC analysis of CHA_2_DS_2_-VASc score and D-dimer in predicting LAAT. LAAT = left atrial appendage thrombus.

Table 2 shows the results of ROC curve analysis. The cutoff points of CHA_2_DS_2_-VASc score, D-dimer and anteroposterior diameter of LA were 1.5, 255.5 μg/L and 49.5 mm, respectively, to predict LAAT. ROC curves are shown in Figures 1 and 2.

**Table 2:** ROC curve analysis to predict the cut-off values (CHA_2_DS_2_-VASc scores, D-dimer and LA diameters) of LAAT

Variables	Cut-off	Sensitivity	Specificity	AUC	95% CI	*P* value
CHA_2_DS_2_-VASc score	1.5	1	0.39	0.73	(0.65–0.81)	<0.001
D-dimer, μg/L	255.5	0.46	0.90	0.67	(0.54–0.80)	0.006
LA anteroposterior diameter, mm	4.95	0.50	0.92	0.78	(0.68–0.88)	<0.001

AUC, area under the curve; CI, confidence interval; LA, left atrium.

### 3.3 Logistic regression analysis

According to the cutoff values, the normal D-dimer group (normal group) was defined as the value of D-dimer <255.5 μg/L, and the elevated D-dimer group (elevated group) was defined as the D-dimer value ≥255.5 μg/L. The smaller LA group was defined as LA < 49.5 mm, and larger LA group was defined as LA ≥ 49.5 mm. Given the low specificity or sensitivity of cutoff points of CHA_2_DS_2_-VASc score (specificity 0.39) and D-dimer (sensitivity 0.46), we did not classify CHA_2_DS_2_-VASc score and D-dimer according to cutoff points for further analysis. Logistic regression showed that larger LA anteroposterior diameter (≥49.5 mm) and non-paroxysmal AF were associated with higher risks of LAAT after adjusting for other confounders (OR = 7.28, 95% CI: 2.36–22.47; OR = 8.89, 95% CI: 2.33–33.99, respectively) (Table 3).

**Table 3:** Logistic regression analysis to evaluate risk factors of LAAT

Variables	B	SE	Wald	Adjusted OR (95% CI)	*P* value
D-dimer (elevated/normal)	1.01	0.59	2.92	2.75 (0.86–8.75)	0.088
LA diameter (larger/smaller)	1.99	0.58	11.91	7.28 (2.36–22.47)	0.001
AF type (non-paroxysmal/paroxysmal)	1.55	0.46	10.21	8.89 (2.33–33.99)	0.001

B, regression coefficient; SE, standard error; OR, odds ratio; CI, confidence interval; LA, left atrium; AF, atrial fibrillation.

### 3.4 Proportions of LAAT in patients with risk factors

The proportions of LAAT in patients with larger LA (LA anteroposterior diameter ≥49.5 mm), non-paroxysmal AF, and both larger LA and non-paroxysmal AF were 30% (12/40), 15.2% (17/112) and 39.1% (9/23), respectively.

## 4 Discussion

In this retrospective study, we performed comparable analysis, ROC curve analysis and logistic regression analysis, and found that enlarged LA anteroposterior diameter (≥49.5 mm) and non-paroxysmal AF were independently associated with higher risks of LAAT (OR = 7.28, 95% CI: 2.36–22.47; OR = 8.89, 95% CI: 2.33–33.99, respectively).

The susceptibility of LAA to thrombosis is related to its anatomical structure, with a dead end and abundant comb muscle [10]. Once LAAT dislodges, serious systemic embolisms, such as cerebral artery embolization, renal artery embolization, mesenteric artery embolization, coronary artery embolization, limb artery embolization, etc., may occur [11]. Therefore, it is important to find potential risk factors of LAAT. Several scoring systems are used to assess the risk of thromboembolism in non-valvular AF patients [12,13,14,15], and to guide anticoagulation strategy. Among them, CHA_2_DS_2_-VASc (congestive heart failure, hypertension, age ≥75 [doubled], diabetes mellitus, prior stroke or transient ischemic attack [doubled], vascular disease, age 65–74, female) is the most powerful scoring system for the prediction of stroke [16]. Uz et al. reviewed 309 non-valvular patients who had undergone TEE, and calculated the CHA2DS2-VASc score for each patient. They found that the risk of LAAT increased with increasing CHA2DS2-VASc score. The CHA2DS2-VASc score was an independent risk factor for LAAT (OR 3.26, 95% CI: 2.3–4.65; *p* = 0.001) in multivariate logistic analysis [17]. We also found that patients with LAAT had higher CHA_2_DS_2_-VASc scores compared with patients without LAAT. However, no exact cutoff point of CHA_2_DS_2_-VASc score was used to predict the risk of LAAT because of the low specificity (0.39) in this study.

Blood biomarkers were found to be associated with the presence of LAAT. For example, a study by Habara showed diagnostic discrimination with D-dimer, with an OR of 97.6 (95% CI: 17.3–595.8) for LAAT and a 97% negative predictive value [18]. In our study, patients in the LAAT group had higher value of D-dimer (180.0 vs 90.0 μg/L, *p* = 0.003) compared with the non-LAAT group. However, no exact cutoff point was used to predict the risk of LAAT because of low sensitivity (0.46). The difference was that the proportion of recent embolic events 2 weeks before TEE was high (23%) in the previous study.

By analyzing the clinical data of patients with non-valvular AF who underwent TEE, we found that enlarged LA anteroposterior diameter was a risk factor of LAAT in non-valvular AF patients. Scherr et al. included 732 cases referred for catheter ablation of AF. All patients were anti-coagulated for ≥4 weeks prior to the procedure. TEE was performed in all patients within 24 h prior to ablation. A total of 12 patients had LA thrombus (1.6%), and larger LA diameter was found to be associated with LA thrombus (OR = 1.6, 95% CI: 1.1–2.3) [19]. To investigate predictors of LAAT formation in patients with AF, Nishikii-Tachibana studied 543 AF patients who underwent TEE before pulmonary vein isolation. All patients were anti-coagulated with warfarin before ablation and LAATs were observed in 2.1% of patients. Multivariate analysis showed that increased LA volume (>50 mL) was significantly associated with increased prevalence of LAATs [20]. The two studies included patients treated with anticoagulants for ≥3 weeks before the ablation. In this study, we excluded patients who had received anticoagulant therapy more than 3 weeks before TEE tests to avoid the effects of anticoagulants on LAAT. However, results of the above studies were similar to our study, and we also found that enlarged LA increased LAAT risk. Our data revealed that enlarged LA (anteroposterior diameter ≥49.5 mm) was an independent risk factor of LAAT (OR = 7.28, *p* < 0.001). The exact mechanism by which enlarged LA increases LAAT risk remains unclear. Generally, enlargement of the LA represents remodeling of the atrial structure, and severe LA remodeling can lead to deterioration of atrial mechanical function (loss of atrial contractile force) resulting in blood flow stagnation and thrombosis in the atrium.

In addition, our study revealed that patients with non-paroxysmal AF were associated with a higher risk of LAAT compared with patients with paroxysmal AF (OR = 8.89, *p* < 0.001). This result was different from traditional clinical research in observing the risk of TE events in AF patients. In 2000, Hart et al. published a cohort study comparing 460 subjects with intermittent AF with 1,552 sustained AF patients treated with aspirin, and followed-up for a mean of 2 years. Analysis showed that the annualized rate of ischemic stroke was similar between those with intermittent (3.2%) and sustained AF (3.3%) [21]. Diagnosis of intermittent AF required at least two electrocardiogram-documented episodes before entry, and no dynamic electrocardiogram (Holter) was used. This design was easier to include intermittent patients with relatively high burden of AF, and may be the source of inconsistent results. Similar to our finding, Scherr et al. observed that patients who were in AF at the time of TEE were more likely to have thrombus compared with all other patients (2.9% vs 0.7%; *p* = 0.03) [19]. The atrium shrinks 350–600 beats per minute every day during AF, and prolonged rapid shrinkage may lead to loss of LA function, resulting in LAAT. Therefore, patients with persistent AF can easily form LAAT compared with patients with paroxysmal AF because of the different durations [22].

Finding the risk factors for LAAT is of great significance to identify the high-risk patients. The proportions of LAAT in patients with larger LA (LA anteroposterior diameter ≥49.5 mm), non-paroxysmal AF, and both larger LA and non-paroxysmal AF were 30% (12/40), 15.2% (17/112) and 39.1% (9/23), respectively, before anticoagulant therapy. Therefore, we recommend that TEE should be performed if risk factors are detected (LA anteroposterior diameter ≥49.5 mm or/and non-paroxysmal AF) for evaluation of LAAT.

## 5 Conclusion

Enlarged LA (anteroposterior diameter ≥49.5 mm) and non-paroxysmal AF were independent risk factors of LAAT in non-valvular AF patients.
